# Metabolic engineering of a xylose pathway for biotechnological production of glycolate in *Escherichia coli*

**DOI:** 10.1186/s12934-018-0900-4

**Published:** 2018-03-28

**Authors:** Min Liu, Yamei Ding, Mo Xian, Guang Zhao

**Affiliations:** 1 CAS Key Laboratory of Biobased Materials, Qingdao Institute of Bioenergy and Bioprocess Technology, Chinese Academy of Siences, Qingdao, 266101 China; 2 Shandong Provincial Key Laboratory of Synthetic Biology, Qingdao, 266101 China; 3 Institute of Oceanology, Chinese Academy of Sciences, Qingdao, 266071 China

**Keywords:** Xylose, Glycolate, Glycolaldehyde, Glyoxylate, *Escherichia coli*

## Abstract

**Background:**

Glycolate is a valuable chemical with extensive applications in many different fields. The traditional methods to synthesize glycolate are quite expensive and toxic. So, the biotechnological production of glycolate from sustainable feedstocks is of interest for its potential economic and environmental advantages. d-Xylose is the second most abundant sugar in nature and accounts for 18–30% of sugar in lignocellulose. New routes for the conversion of xylose to glycolate were explored.

**Results:**

Overexpression of *aceA* and *ghrA* and deletion of *aceB* in *Escherichia coli* were examined for glycolate production from xylose, but the conversion was initially ineffective. Then, a new route for glycolate production was established in *E*. *coli* by introducing NAD^+^-dependent xylose dehydrogenase (*xdh*) and xylonolactonase (*xylC*) from *Caulobacter crescentus*. The constructed engineered strain Q2562 produced 28.82 ± 0.56 g/L glycolate from xylose with 0.60 ± 0.01 g/L/h productivity and 0.38 ± 0.07 g/g xylose yield. However, 27.18 ± 2.13 g/L acetate was accumulated after fermentation. Deletions of *iclR* and *ackA* were used to overcome the acetate excretion. An *ackA* knockout resulted in about 66% decrease in acetate formation. The final engineered strain Q2742 produced 43.60 ± 1.22 g/L glycolate, with 0.91 ± 0.02 g/L/h productivity and 0.46 ± 0.03 g/g xylose yield.

**Conclusions:**

A new route for glycolate production from xylose was established, and an engineered strain Q2742 was constructed from this new explored pathway. The engineering strain showed the highest reported productivity of glycolate to date. This research opened up a new prospect for bio-refinery of xylose and an alternative choice for industrial production of glycolate.

## Background

Glycolate is a notable α-hydroxy acid containing both alcohol and carboxyl groups [1]. Its properties make glycolate ideal for a broad spectrum of applications. It can be used as skincare product in the cosmetic industry, as rinsing agents in dyeing and tanning industry and as precursors in biopolymers synthesis [2–4]. The polymer of glycolate is an excellent packaging material due to the properties of gas-barrier and mechanical strength [5]. Glycolate is also used to be polymerized with lactic acid for medical applications [6]. With increasing demands, the glycolate market is expected to reach USD 277.8 million in 2020 (http://www.grandviewresearch.com/press-release/global-glycolic-acid-market).

Currently, glycolate is manufactured through two traditional methods. One is produced by chemical carbonylation of formaldehyde under high-temperature, high-pressure condition [7]. The other is produced from ethylene glycol by enzyme-catalyzed oxidation reaction [4], or from glycolonitrile by hydrolyzation reaction [8]. However, precursors of all the above reactions are quite expensive or toxic.

The biotechnological production of glycolate from sustainable feedstock, such as lignocellulose would be more economically feasible and environmental friendly [9–11]. d-glucose and d-xylose are the most abundant constituents of lignocellulose, which are principally derived from cellulose and hemicellulose, respectively [12]. d-glucose was easily metabolized by most microorganisms, and for this reason, metabolic engineering for glycolate production was mainly concentrated on the glyoxylate bypass with glucose as the carbon source [13–15]. However, xylose is the second most abundant sugar in nature, and accounts for 18–30% of sugar in lignocellulose [16]. Therefore, the capability to efficiently metabolize xylose is a desirable attribute of microorganisms for optimizing the economics of lignocellulose-based bio-refinery processes. In fact, only a small fraction of microorganisms has the native metabolic pathway of xylose [17]. d-xylose is initially converted to d-xylulose by the catalysis of different enzymes in microorganisms. Bacteria like *Escherichia coli* generally employ a one-step pathway using xylose isomerase (XI) enzyme, whereas yeasts and mycelial fungi need two enzymatic–catalytic steps by reduction reaction of d-xylose reductase (XR) and oxidation reaction of xylitol dehydrogenase (XDH) [18, 19]. d-xylulose is further metabolized to d-xylulose-5-phosphate and then directed to pentose phosphate pathway (PPP) in both bacteria and fungi [20]. Recently, researchers reported a new metabolism pathway of xylose through yielding α-ketoglutarate in the oligotrophic freshwater bacterium *Caulobacter crescentus* [21]. This novel pathway was initiated by the enzymes of NAD^+^-dependent xylose dehydrogenase (Xdh) and xylonolactonase (XylC) [21, 22]. All enzymes of this xylose degradation pathway in *C. crescentus* were encoded by the *xyl* operon and expressed by xylose induction [23, 24].

**Fig. 1:**
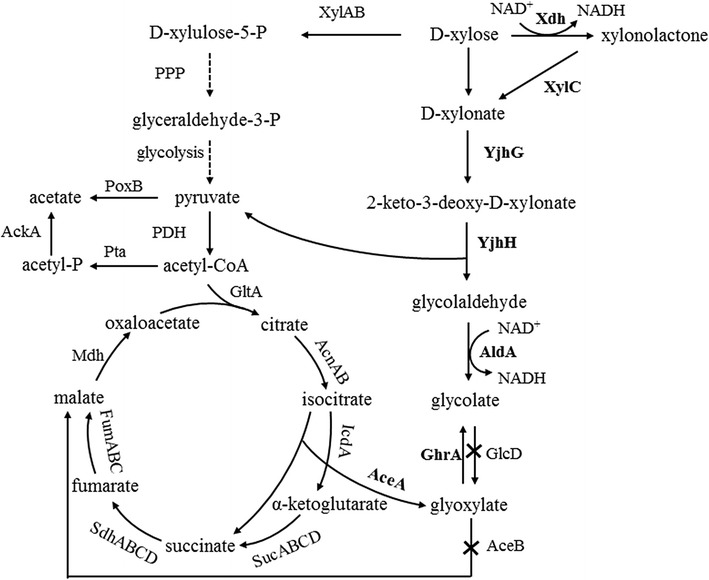
The metabolic pathway for production of glycolate from xylose in glycolaldehyde oxidation pathway and glyoxylate reduction pathway. Enzymes associated with the reactions were showed and the over expressions of which were marked in bold. d-xylose was converted to d-xylonate by xylose dehydrogenase (*xdh*) and xylonolactonase (*xylC*) from *C. crescentus*. All other genes were from the native *E. coli* BL21(DE3)

*Escherichia coli* uses the pentose phosphate pathway for the metabolism of xylose. An important intermediate acetyl-CoA is produced by the pentose phosphate pathway and the glycolysis. Then it is metabolized by the tricarboxylic acid (TCA) cycle (Fig. 1).

The intermediate isocitrate is not only converted to succinate by isocitrate dehydrogenase (*icd*) and α-ketoglutarate dehydrogenase (*suc*) in TCA cycle, but also is directly split into succinate and glyoxylate by isocitrate lyase (*aceA*) in glyoxylate bypass without CO_2_ emission. Finally, glyoxylate is reduced to glycolate by the bifunctional glyoxylate/hydroxypyruvate reductase (*ghrA*). The biosynthesis of glycolate in some previous studies was concentrated on glyoxylate bypass from glucose [13–15]. The previous results demonstrated that increased expression of *aceA* and *ghrA* and decreased expression of *aceB* showed positive effects on glycolate production with glucose as the carbon. *E. coli* have the native xylose assimilation pathway, and glycolate may also be produced from xylose via reduction of the intermediate glyoxylate, which is provided by the glyoxylate bypass. In order to verify this hypothesis, the glyoxylate reduction pathway was constructed and determined for the production of glycolate with xylose as the carbon. Besides, xylose can be oxidized to xylonate with generating one NADH in xylose assimilation pathway of *C. crescentus*. The latter is directly metabolized to pyruvate and glycolaldehyde via the dehydration and aldolytic cleavage (Fig. 1). Compared with the *E. coli* native XI pathway, this route shortens the xylose assimilation process which may improve the efficiency theoretically. So, a novel route was also designed for glycolate production in *E. coli* by introducing the xylose assimilation pathway of *C. crescentus*. Glycolate was gained from oxidation of the intermediate glycolaldehyde in this route, named the glycolaldehyde oxidation pathway. Meanwhile, the glyoxylate reduction pathway and the glycolaldehyde oxidation pathway were engineered simultaneously for determining and discussing the effects of the combination on glycolate production. This research may opened up a new prospect for bio-refining of xylose and improve the large scale production of glycolate.

## Results and discussion

### Production of glycolate from glyoxylate reduction pathway

*Escherichia coli* strains have the native xylose metabolism pathway. d-xylose was metabolized to glyoxylate catalyzed by a series of enzymes and further reduced to glycolate by GhrA. The glyoxylate reduction pathway for glycolate production was constructed. The coding regions of *E. coli* native genes *aceA* and *ghrA* were cloned to pACYCDuet1 and pETDuet1, respectively. The two recombinant plasmids pACYCDuet1-*aceA* (pLMa1) and pETDuet1-*ghrA* (pLMe1) were transformed to *E. coli* BL21(DE3), creating the engineered strain Q2728. The proteins of AceA and GhrA showed proper expressions as compared with the control strain *E. coli* BL21(DE3) harboring the empty plasmids pACYCDuet1 and pETDuet1 by gel electrophoresis analysis. The engineered strain Q2728 and wild-type *E. coli* BL21(DE3) were cultured with 15 g/L xylose as the sole carbon source. After 24 h, glycolate was not detected in *E. coli* BL21(DE3), suggesting that cells could not accumulate glycolate via the unmodified glyoxylate bypass (data was not shown). The engineered strain Q2728 accumulated 0.28 ± 0.01 g/L glycolate at a yield of 0.04 ± 0.01 g/g xylose (Table 1). In order to block the flux from glyoxylate to malate in glyoxylate bypass, the malate synthase (*aceB*) gene was knocked out to generate *aceB* mutant strain Q2589. The above recombinant plasmids pLMe1 and pLMa1 were transformed to Q2589, creating the engineered strain Q2705. As shown in Table 1, the glycolate production of Q2705 was improved to 0.51 ± 0.11 g/L, showing 1.8 times higher than that of Q2728. However, the xylose consumption of Q2705 also showed about 1.5 times higher as compared to Q2728. Through calculation, the glycolate yield of Q2705 was 0.05 ± 0.03 g/g xylose, similar to the value of Q2728. So, knockout of *aceB* had no significant improvement of the glycolate yield.

**Table 1:** Production of glycolate after 24 h of IPTG induction under Erlenmeyer flasks cultivation on minimal medium with 15 g/L d-xylose

Strains	Cell biomass (g_CDW_/L)	Cell yield (g/g xylose)	Glycolate concentration (g/L)	Glycolate yield (g/g xylose)	Acetate concentration (g/L)	Acetate yield (g/g xylose)
Q2728	1.05 ± 0.01	0.16 ± 0.07	0.28 ± 0.01	0.04 ± 0.01	0.66 ± 0.01	0.10 ± 0.02
Q2705	1.12 ± 0.04	0.14 ± 0.02	0.51 ± 0.11	0.05 ± 0.03	1.33 ± 0.15	0.16 ± 0.05
Q2562	1.03 ± 0.16	0.18 ± 0.09	2.53 ± 0.08	0.44 ± 0.10	0.95 ± 0.18	0.17 ± 0.04
Q2590	1.09 ± 0.13	0.24 ± 0.04	1.68 ± 0.09	0.37 ± 0.06	0.86 ± 0.13	0.19 ± 0.01
Q2725	0.99 ± 0.08	0.15 ± 0.11	2.65 ± 0.12	0.41 ± 0.14	0.83 ± 0.14	0.13 ± 0.06
Q2726	0.98 ± 0.15	0.16 ± 0.01	2.54 ± 0.03	0.41 ± 0.12	0.91 ± 0.11	0.15 ± 0.10
Q2727	1.02 ± 0.07	0.17 ± 0.05	2.61 ± 0.15	0.44 ± 0.08	0.84 ± 0.02	0.14 ± 0.07
Q2729	1.11 ± 0.05	0.18 ± 0.02	2.72 ± 0.11	0.43 ± 0.05	1.07 ± 0.07	0.17 ± 0.01

Data were averages of triplicate experiments, and errors represent standard deviation

A few approaches that engineered glyoxylate bypass for the production of glycolate from glucose in different microorganisms have been reported [13–15]. For examples, deletion of *aceB* and heterologous expression of *ghrA* from *E. coli* resulted in a titer of 4 g/L glycolate in *Corynebacterium glutamicum* with glucose as the sole carbon source. When the translation start codon ATG of isocitrate dehydrogenase (*icd*) was replaced by GTG, the production of glycolate was further increased to 5.3 ± 0.1 g/L [15]. A *C. glutamicum* strain was capable to produce glycolate by engineered glyoxylate bypass with glucose as the carbon source. The engineered *E. coli* strain EYX-2 had the highest reported glycolate titer of 56.44 g/L with 0.52 g/g glucose yield under 120 h fed-batch cultivation [14]. However, in our study, the titer of glycolate was only 0.51 ± 0.11 g/L via expression of *aceA* and *ghrA* and deletion of *aceB*. This titer was significantly lower than the previous results with glucose as carbon via the similar engineering. As shown in Fig. 1, the intermediate isocitrate can be both converted to α-ketoglutarate by isocitrate dehydrogenase (*icd*) and to glyoxylate by isocitrate lyase (*aceA*). The above two previous studies were all included the regulation of α-ketoglutarate branch. Therefore, our constructed route may redirect the carbon fluxes to glyoxylate synthesis insufficiently due to the α-ketoglutarate branch. Besides, the different carbon supply in our study may yield the different results as compared to the previous studies with glucose as the carbon even if the similar metabolic engineering is employed. The exact mechanism will be unravelled based on the metabonomic researches in future.

### Production of glycolate from glycolaldehyde oxidation pathway

In this work, a new direct conversion from xylose to glycolate in an engineered *E. coli* was explored by introducing the xylose degradation pathway of *C. crescentus*. This new route for the bio-production of glycolate mainly consists of three parts (Fig. 1). Firstly, d-xylose was metabolized to d-xylonate by the catalysis of two heterogeneous enzymes Xdh and XylC from *C. crescentus*. Subsequently, d-xylonate was converted to glycolaldehyde by the *E. coli* native enzymes of xylonate dehydratase (*yjhG*) and 2-keto-3-deoxy-d-xylonate aldolase (*yjhH*) which has been identified in the earlier study [25, 26]. Finally, glycolaldehyde was oxidized to glycolate by the catalysis of aldehyde dehydrogenase (*aldA*). Overall, our constructed pathway was significantly different from the previous reported pathways for glycolate production from xylose [27, 28]. Stephanopoulos et al. demonstrated the assimilation of xylose via isomerization to xylulose, epimerization of xylulose to ribulose, and phosphorylation of the latter to yield ribulose-1-P, which was followed by an aldolytic cleavage to obtain glycolaldehyde and DHAP [27]. This pathway was similar to the work of Walther et al. In the constructed pathway of Walther et al. xylose was also assimilated to xylulose via isomerization. Then, xylulose was directly phosphorylated to xylulose-1-P and cleaved to glycolaldehyde and DHAP [28]. Though these two pathways had the same stoichiometry, the former additionally employed the synthetic xylulose epimerase reaction, which may complicate implementation of the pathway because another enzymatic activity has to be identified and optimized by protein engineering. However, in our designed pathway, d-xylose was oxidized to d-xylonate via two heterogeneous enzymes Xdh and XylC from *C. crescentus*. d-xylonate was then dehydrated to 2-dehydro-3-deoxy-d-xylonate, followed by an aldolytic cleavage to yield glycolaldehyde and pyruvate. The xylose assimilation in our designed pathway could produce one NADH via xylose oxidation step and did not depend on a phosphorylation step, which consumed one additional ATP per molecule of xylose. So, our pathway may have lower energy consumption. These advantages will be potential for the large-scale production of glycolate.

**Fig. 2:**
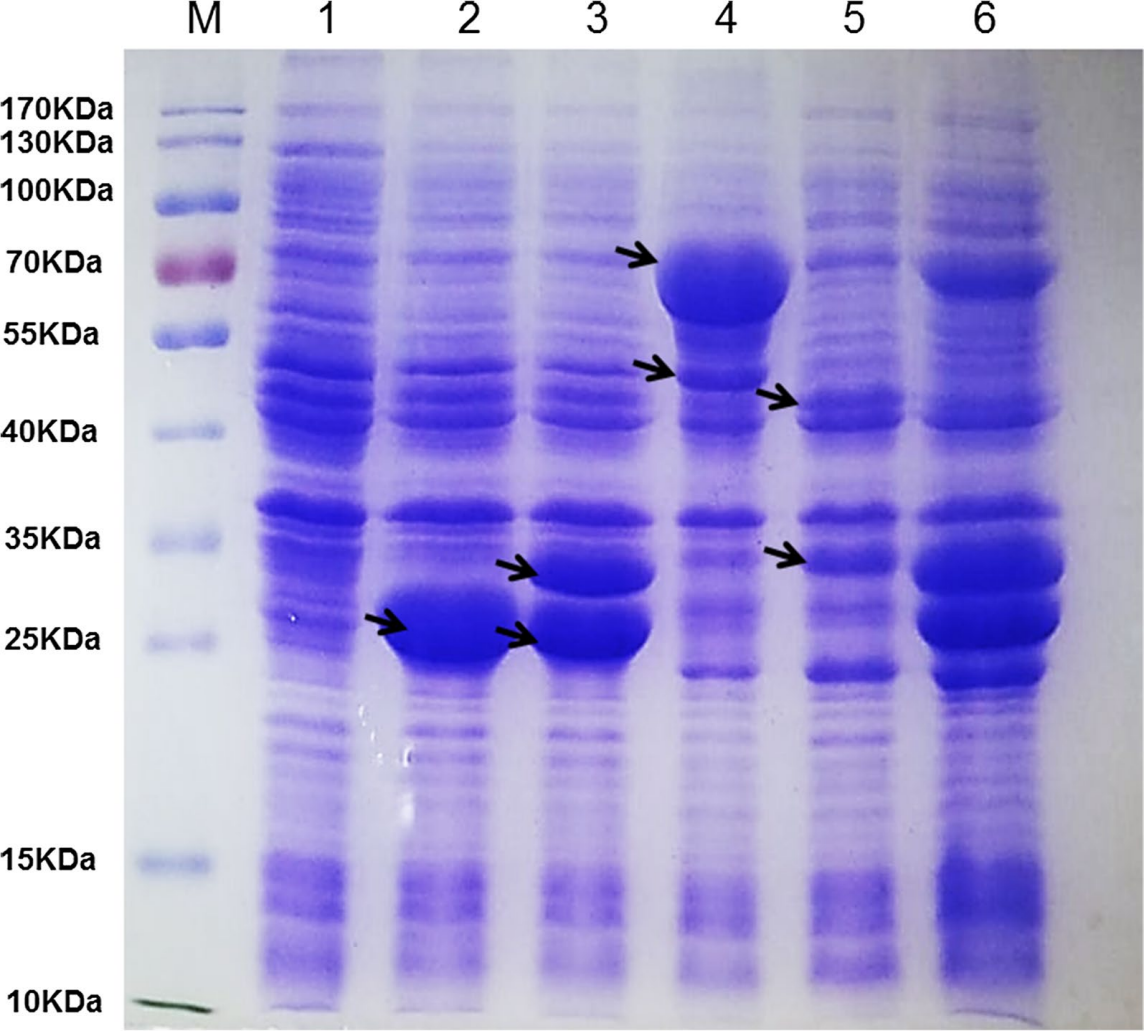
Coomassie brilliant blue-stained SDS-PAGE analysis of recombinant proteins from different constructs. Lane M, protein molecular weight marker; lane 1, crude cells extracts from *E. coli* BL21(DE3)/pETDuet1/pACYCDuet1; lane 2, crude cells extracts from *E. coli* BL21(DE3) strain harboring pETDuet1-*yjhH*; lane 3, crude cells extracts from *E. coli* BL21(DE3) strain harboring pETDuet1-*xylC*-*xdh*; lane 4, crude cells extracts from *E. coli* BL21(DE3) strain harboring pACYCDuet1-*aldA*-*yjhG*; lane 5, crude cells extracts from *E. coli* BL21(DE3) strain harboring pETDuet1-*ghrA and* pACYCDuet1-*aceA*; lane 6, crude cells extracts from *E. coli* BL21(DE3) strain harboring pETDuet1-*yjhH*-*xylC*-*ghrA*-*xdh* and pACYCDuet1-*aldA*-*aceA*-*yjhG*. The position corresponding to the overexpressed genes were indicated by an arrow

The engineered *E. coli* strain Q2562 was constructed by transformation of the two recombinant plasmids pLMe2 and pLMa2. The gel electrophoresis analysis was carried out for detecting the expressions of all the introduced genes. Figure 2 showed patterns of protein samples visualized by Coomassie Brilliant Blue staining. We noted distinct bands of the expected sizes from crude extracts of the recombinant strains when compared with the control strain. All the distinct bands of the proteins YjhH, XylC, Xdh, YjhG, AldA were found in Fig. 2 and confirmed that the introduced genes in different recombinant plasmids were expressed obviously.

After cultivation of 24 h under shake-flask experiments, the glycolate production of Q2562 was 2.53 ± 0.08 g/L at the yield of 0.44 ± 0.10 g/g xylose, showing about ten times higher than those of Q2728 from the glyoxylate reduction pathway.

The cell biomass of the culture was 1.03 ± 0.16 g_CDW_/L, similar to the cell biomass as compared to that of Q2728 and Q2705 (Table 1). Considering all these results, a new route for the bio-production of glycolate from xylose was successfully established in *E. coli.* D-xylose was directly metabolized to D-xylonate by heterogenous enzymes Xdh and XylC from *C. crescentus* and then converted to glycolate by the native enzymes from *E. coli*. This research opened up a new prospect for bio-refinery of xylose and a new technology for the large-scale production of glycolate.

### Production of glycolate from the two-combined pathway

In this new established glycolaldehyde oxidation pathway for the production of glycolate, the intermediate 2-keto-3-deoxy-d-xylonate was split to glycolaldehyde and pyruvate catalyzed by YjhH (Fig. 1). The yield of glycolate may be decreased due to the distribution of carbon flux to pyruvate. However, pyruvate could be converted to glycolate by a series of enzyme-catalyzed reactions from glyoxylate reduction pathway. Therefore, the glycolate yields may be improved by combining the glycolaldehyde oxidation pathway and glyoxylate reduction pathway. Genetic modifications of *aceB* deletion, *aceA* over-expression and *ghrA* over-expression were independently incorporated into strain Q2562, yielding new strains of Q2590, Q2725 and Q2726. The three strains were also cultivated in shake-flask with d-xylose as the sole carbon source. Surprisingly, Q2590 showed lower glycolate production and yield than those of Q2562. This result seems likely that the reduction of malate formation by *aceB* deletion reduces the formation of oxaloacetate which is necessary for the formation of isocitrate in the first place (Fig. 1). While Q2725 and Q2726 showed the similar glycolate productions and yields to those of Q2562 (Table 1). Due to the negative effects of *aceB* knockout on glycolate production, over-expressions of *aceA* and *ghrA* were chosen to incorporate into the strain Q2562 simultaneously, yielding a new strain Q2729. The engineered strain Q2729 reached 2.72 ± 0.11 g/L glycolate, higher than the value 2.53 ± 0.08 g/L of Q2562 (Table 1). However, the glycolate yield of Q2729 was similar to that of Q2562. Consistent with the above results, the glycolate production from xylose in glyoxylate reduction pathway was initially ineffective. Therefore, the glycolate productions and yields from the two-combined pathway were not significantly improved than those from the glycolaldehyde oxidation pathway.

The mutual transformation of glyoxylate and glycolate occurres in *E. coli* strains. Glyoxylate was reduced to glycolate by glyoxylate reductase (*ghrA*), and in turn, glycolate was oxidated to glyoxylate by glycolate oxidase (*glcD*) (Fig. 1). In order to avoid the conversion from glycolate to glyoxylate, the *glcD* gene was knocked out from Q2562, yielding strain Q2727. As shown in Table 1, the engineered strain Q2727 reached 2.61 ± 0.15 g/L glycolate, slightly higher than the value of 2.53 ± 0.08 g/L glycolate of Q2562. These results indicated that the reaction from glycolate to glyoxylate by GlcD had little effects on glycolate production.

### Production of glycolate under fed-batch cultivation

**Fig. 3:**
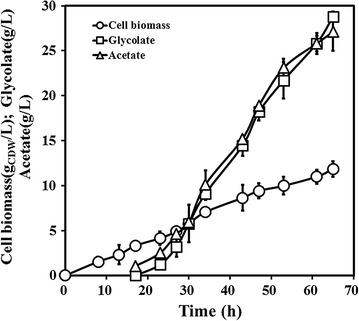
Time profiles for the cell biomass, glycolate production and acetate production of the engineered strain *E. coli* BL21(DE3)/pLMe2/pLMa2(Q2562) under fed-batch cultivation. Culture was performed in a 3 L laboratory fermenter containing 1 L of fermentation medium. Error bars represent ± standard deviation from the mean

In order to test the suitability of engineered strains for the larger-scale production of glycolate, the fed-batch cultivation was performed based on the results from the shake-flask cultures. The glycolate productions and yields of the engineered strains from two-combined pathway showed no obvious improvement than those of the strain Q2562 from glycolaldehyde oxidation pathway. So, the engineered strain Q2562 was performed for the fed-batch cultivation in a 3 L-scale laboratory fermenter. The OD_600_ and the concentrations of residual xylose, glycolate and acetate were monitored during the course of cultivation. The residual xylose concentration was controlled below 5 g/L. Figure 3 showed the time profiles of cell biomass, glycolate concentration and acetate concentration during the whole fermentation period, and all the three values increased gradually with time. After fermentation, the glycolate production of Q2562 was 28.82 ± 0.56 g/L and the glycolate yield was 0.38 ± 0.07 g/g xylose (Table 2). Importantly, the productivity of Q2562 was 0.60 ± 0.01 g/L/h, significantly higher than that of the value 0.47 g/L/h of the engineered *E. coli* strain EYX-2 which had the reported highest glycolate production from glucose. It was speculated that the strain Q2562 from glycolaldehyde oxidation pathway has the potential for large scale production of glycolate.

**Table 2:** Production of glycolate after 48 h of IPTG induction under fed-batch cultivation on minimal medium with d-xylose

Strains	Cell biomass (g_CDW_/L)	Cell yield (g_CDW_/g xylose)	Glycolate concentration (g/L)	Glycolate yield (g/g xylose)	Acetate concentration (g/L)	Acetate yield (g/g xylose)	Glycolate productivity (g/L/h)
Q2562	11.84 ± 0.85	0.16 ± 0.02	28.82 ± 0.56	0.38 ± 0.07	27.18 ± 2.13	0.36 ± 0.03	0.60 ± 0.01
Q2741	9.54 ± 0.76	0.11 ± 0.06	38.21 ± 0.86	0.42 ± 0.12	21.86 ± 1.31	0.24 ± 0.05	0.79 ± 0.01
Q2742	9.25 ± 0.32	0.10 ± 0.01	43.60 ± 1.22	0.46 ± 0.03	9.08 ± 0.87	0.10 ± 0.10	0.91 ± 0.02

Data were averages of triplicate experiments, and errors represent standard deviation

To our surprise, Q2562 accumulated 27.18 ± 2.13 g/L acetate after cultivation. Acetate overflow was also occurred in fed-batch fermentation of engineered strain EYX-2, which was also about 20 g/L [14]. So, acetate overflow is one of the major problems for the large scale production of glycolate both from glucose to xylose. Acetate was produced from pyruvate by pyruvate oxidase (*poxB*) and produced from acetyl-CoA by phosphotransacetylase-acetate kinase (*pta*-*ackA*), respectively (Fig. 1). Approaches for overcoming acetate overflow may be beneficial for improving the glycolate production and xylose efficiency.

### Overcoming acetate formation through *iclR* and *ackA* deletion

**Fig. 4:**
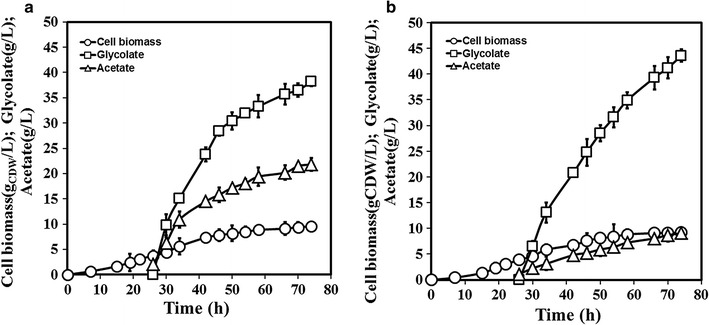
Knockout of *iclR* and *ackA* for overcoming acetate excretion. **a** Time profiles for the cell biomass, glycolate production and acetate production of *iclR* konckout strain *E. coli* BL21(DE3) *iclR*::*Kan*/pLMe2/pLMa2 (Q2741) under fed-batch cultivation. **b** Time profiles for the cell biomass, glycolate production and acetate production of the *ackA* knockout strain *E. coli* BL21(DE3) *ackA*::*Kan*/pLMe2/pLMa2 (Q2742) under fed-batch cultivation. Strains were cultivated in a 3 L laboratory fermenter containing 1 L of fermentation medium. Error bars represent ± standard deviation from the mean

Acetate secretion has adverse effects on the cell growth, the macromolecules synthesis and the desirable metabolites formation [29, 30]. In order to overcome the acetate overflow, deletions of *iclR* and *ackA* were carried out. IclR (isocitrate lyase regulator) is a transcriptional regulator that represses gene expression of *aceBAK* operon. The *aceBAK* operon is responsible for coding the glyoxylate bypass enzymes of malate synthase (*aceB*), isocitrate lyase (*aceA*) and isocitrate dehydrogenase kinase/phosphatase (*aceK*) [31–33]. Glyoxylate bypass is the main pathway for acetate assimilation, and decreasing the gene expression of *iclR* may activate glycolate cycle and decrease acetate accumulation. AckA (acetate kinase) belongs to the acetate synthetic pathway. Deletion of *ackA* is a direct method to affect acetate formation [34]. The *iclR* and *ackA* were knocked out, yielding *iclR* mutant Q2280 and *ackA* mutant Q1951. The recombinant plasmids pLMe2 and pLMa2 were simultaneously transformed to Q2280 and Q1951, creating the engineered strain Q2741 and Q2742, respectively. Both the Q2741 and Q2742 strains were cultivated under fed-batch cultivation in a 3 L-scale laboratory fermenter. The results showed that knockout of *iclR* and *ackA* significantly improved the glycolate productions, productivities and yields (Table 2 and Fig. 4). The engineered strain Q2741 produced 38.21 ± 0.86 g/L glycolate, with 0.79 ± 0.01 g/L/h productivity and 0.42 ± 0.12 g/g xylose yield. Knockout of *iclR* improved the glycolate production by 1.32-fold than that of Q2562. Moreover, knockout of *ackA* showed better results for the above aspects. The engineered strain Q2742 produced 43.60 ± 1.22 g/L glycolate, showing 1.51-fold higher than that of Q2562. The productivity and yield reached 0.91 ± 0.02 g/L/h and 0.46 ± 0.03 g/g, respectively. This is the highest reported production of glycolate from xylose to the highest reported productivity to date.

In our designed glycolaldehyde oxidation pathway, xylose was metabolized to 2-keto-3-deoxy-d-xylonate and then cleaved to glycolaldehyde and pyruvate. Therefore, 1 mol glycolate could be produced from 1 mol xylose via the oxidation of intermediate glycolaldehyde. As this calculation, the maximum theoretical yield was about 0.51 g/g xylose. Obviously, the yield of engineering strain Q2742 approximated to the maximum theoretical yield through overcoming acetate formation. Compared to the work of Stephanopoulos et al. an engineered strain GA-10 produced 40 g/L glycolate, which presented the production comparable to that obtained by our study [27]. However, the productivity of our engineered strain Q2742 showed two times higher than that of the engineered strain GA-10. The increased productivity of the novel pathway may be of interest for the biotechnological production of glycolate.

Knockout of *ackA* showed dramatic effect on overcoming acetate overflow. The acetate concentration of Q2742 was decreased by 66%, from 27.18 ± 2.13 to 9.08 ± 0.87 g/L. It was only decreased by about 20%, from 27.18 ± 2.13 to 21.86 ± 1.31 g/L through *iclR* deletion. IclR is a transcriptional regulator that indirectly regulates acetate metabolism through *aceBAK* operon in glycolate bypass. By contrast, AckA is directly related to the acetate formation. It may be the reason for the phenomenon that *ackA* deletion showed more obvious effect on overcoming acetate overflow than *iclR* deletion. In this new constructed route for the production of glycolate, the intermediate 2-keto-3-deoxy-d-xylonate was split into pyruvate and glycolaldehyde by the catalysis of YjhH. Then the byproduct acetate was produced from pyruvate to its downstream acetyl-CoA (Fig. 1). Overcoming acetate formation through transcription regulations may improve the distribution of carbon flux for glycolate biosynthesis pathway and finally improve the glycolate production. In addition, the final cell biomass of Q2742 was much lower as compared to that of Q2562. The similar cases were also reported in some previous studies, suggesting that modifications of acetate metabolic pathway usually have negative impacts on cell growth [35–37]. A possible explanation is that ATP is released in the process of acetate production by Pta-AckA pathway. Disruption of the Pta-AckA pathway may affect ATP provision for the cell growth. In conclusion, the lower acetate yield and cell yield of Q2742 reflected the fact that cells regulate more carbon flux for glycolate production and it may be proved by the metabolomics research in future.

## Conclusions

In this study, a new route (glycolaldehyde oxidation pathway) for biosynthesis of glycolate was established in *E. coli* by introducing the xylose degradation pathway of *C. crescentus*. d-xylose was directly metabolized to d-xylonate by heterogenous enzymes Xdh and XylC from *C. crescentus* and then converted to glycolate by the native genes from *E. coli*. In addition, *ackA* deletion successfully overcame the serious problem of acetate excretion during the process of glycolate production. The engineered strain Q2742 produced 43.60 ± 1.22 g/L glycolate, with 0.91 ± 0.02 g/L/h productivity and 0.46 ± 0.03 g/g xylose yield under fed-batch cultivation. The acetate concentration of Q2742 was decreased 66% by knocking out of *ackA* gene. In summary, this research opened up a new prospect for bio-refinery of xylose and established a new route for the biosynthesis of glycolate. The engineered strain Q2742 has the capacity for large scale production of glycolate and it could be widely used in industrial applications.

## Methods

### Plasmids and strains construction

Primers used for this study were listed in Table 3, plasmids and strains used in this study were listed in Table 4. *E. coli* DH5α was used for gene cloning and *E. coli* BL21(DE3) was used as the parent for all strain engineering. The *xylC* (GenBank Accession No.: NACL94328) and *xdh* (GenBank Accession No.: NACL94329) genes from *C. crescentus* were cloned into pETDuet1 vector between *EcoR*I, *NotI* and *Nde*I and *Kpn*I sites to generate plasmid pETDuet1-*xylC*-*xdh*. Then *yjhH* (Gene ID: 948825) was cloned into pETDuet1-*xylC*-*xdh* vector between *Nco*I and *EcoR*I sites to create pETDuet1-*yjhH*-*xylC*-*xdh* (pLMe2). The recombinant plasmid pETDuet1-*yjhH* was also constructed under the same promoter to avoid the disturbance of Xdh and XylC with the similar molecular weight in protein expression and gel electrophoresis analysis. Finally, the *ghrA* (Gene ID 946431) was, respectively cloned into pETDuet1 and pLMe2 vectors between the same sites of *NotI* and *Afl*II to create pETDuet1-*ghrA* (LMe1) and pETDuet1-*yjhH*-*xylC*-*ghrA*-*xdh* (pLMe3). The *yjhG* (Gene ID 946829) and *aldA* (Gene ID 945672) were cloned into pACYCDuet1 to generate pACYCDuet1-*aldA*-*yjhG* (pLMa2) vector between *Nde*I, *Xho*I and *Nco*I, *EcoR*I sites. The *aceA* (Gene ID 948517) was cloned into pACYCDuet1 and pLMa2 vectors using the same sites of *EcoR*I and *Hind*III to create pACYCDuet1-*aceA* (pLMa1) and pACYCDuet1-*aldA*-*aceA*-*yjhG* (pLMa3), respectively. The native *E. coli* genes of *yjhH*, *aceA*, *ghrA* and *yjhG* were generated by PCR using primers listed in Table 3. The chromosomal *aceB*, *glcD*, *iclR* and *ackA* genes of *E. coli* BL21 (DE3) strain encoded for malate synthase, glycolate oxidase, isocitrate lyase regulator and acetate kinase were knocked out via P_1_ vir-mediated transduction as previously described [38]. The donor strain JW3974, JW2946, JW3978 and JW2293 were purchased from the Keio collection [39]. The different plasmids were transformed to the corresponding hosts for the productions of glycolate by the glycolaldehyde oxidation pathway and/or glyoxylate reduction pathway.

**Table 3:** Primers used in this study

Name	Description
*yjhH*_F_*Nco*I	GGAATTCCATATGAAAAAATTCAGCGGCATTATTC
*yjhH*_R_*EcoR*I	CCGCTCGAGTCAGACTGGTAAAATGCCCT
*xylC*_F_ *EcoR*I	CCGGAATTCTAATACGACTCACTATAGGGGAATTG
*xylC*_R_*Not*I	AAGGAAAAAAGCGGCCGCTTAAACCAGACGAACTTCGTGCTG
*ghrA*_F_*Not*I	ATTTGCGGCCGCTCGCACAACGCTTTTCGGGA
*ghrA*_R_*Afl*II	CCCACATGTTTAGTAGCCGCGTGCGCGGT
*xdh*_F_*Nde*I	GGGAATTCCATATG TCCTCAGCCATCTATCCC
*xdh*_R_*Kpn*I	CGGGGTACC TCAACGCCAGCCGGCGTCGAT
*aldA*_F_*Nco*I	CCGCCATGGGCATGTCAGTACCCGTTCAACA
*aldA*_R_*EcoR*I	CCGGAATTCTTAAGACTGTAAATAAACCA
*aceA*_F_*EcoR*I	CCGGAATTCCACCACATAACTATGGAGCA
*aceA*_R_*Hind*III	CCCAAGCTTTTAGAACTGCGATTCTTCAG
*yjhG*_F_*Nde*I	GGAATTCCATATG TCTGTTCGCAATATTTTTGC
*yjhG*_R_*XhoI*	CCGCTCGAGTCAGTTTTTATTCATAAAATCGCG
ID-*aceB*_F	GTTATCAACAAGTTATCAAGT
ID-*glcD*_F	TCGATACTCTCTGCAACCAC
ID-*iclR*_F	CATAAAACGGATCGCATAACGC
ID-*ackA*_F	CATAAAACGGATCGCATAACGC
*Kan*_R	GGTGAGATGACAGGAGATCC

**Table 4:** Plasmids and strains used in this study

Plasmids and strains	Description	Source
Plasmids		
pETDuet1	*Amp* ^*r*^ *oriP*_*BR322*_ *lacI* ^*q*^ *P*_*T7*_	Novagen
pACYCDuet1	*Cm* ^*r*^ *oriP*_*15A*_ *lacI* ^*q*^ *P*_*T7*_	Novagen
pLMe1	pETDuet1-*ghrA*	This study
pLMe2	pETDuet1-*yjhH*-*xylC*-*xdh*	This study
pLMe3	pETDuet1-*yjhH*-*xylC*-*ghrA*-*xdh*	This study
pLMa1	pACYCDuet1-*aceA*	This study
pLMa2	pACYCDuet1-*aldA*-*yjhG*	This study
pLMa3	pACYCDuet1-*aldA*-*aceA*-*yjhG*	This study
Strains		
*E. coli* DH5α	F^−^ *supE*44 Δ*lacU*169 (*ϕ*80 *lacZ* Δ*M15*) *hsdR*17 *recA*1 *endA*1 *gyrA*96 *thi*-1 *relA*1	Invitrogen
*E. coli* BL21(DE3)	F^−^ *ompT gal dcm lon hsdSB* (rB^−^ mB^−^) λ(DE3)	Invitrogen
JW3974	*E. coli* BW25113 *aceB*::*kan*	[39]
JW2946	*E. coli* BW25113 *glcD*::*kan*	[39]
JW3978	*E. coli* BW25113 *iclR*::*kan*	[39]
JW2293	*E. coli* BW25113 *ackA*::*kan*	[39]
Q2589	*E. coli* BL21(DE3) *aceB*::*kan*	This study
Q2723	*E. coli* BL21(DE3) *glcD*::*kan*	This study
Q2280	*E. coli* BL21(DE3) *iclR*::*kan*	This study
Q1951	*E. coli* BL21(DE3) *ackA*::*kan*	This study
Q2728	*E. coli* BL21(DE3)/pLMe1/pLMa1	This study
Q2705	*E. coli* BL21(DE3) *aceB*::*kan*/pLMe1/pLMa1	This study
Q2562	*E. coli* BL21(DE3)/pLMe2/pLMa2	This study
Q2590	*E. coli* BL21(DE3) *aceB*::*kan*/pLMe2/pLMa2	This study
Q2725	*E. coli* BL21(DE3)/pLMe2/pLMa3	This study
Q2726	*E. coli* BL21(DE3)/pLMe3/pLMa2	This study
Q2727	*E. coli* BL21(DE3) *glcD*::*kan*/pLMe2/pLMa2	This study
Q2729	*E. coli* BL21(DE3)/pLMe3/pLMa3	This study
Q2741	*E. coli* BL21(DE3) *iclR*::*kan*/pLMe2/pLMa2	This study
Q2742	*E. coli* BL21(DE3) *ackA*::*kan*/pLMe2/pLMa2	This study

### Protein expression and gel electrophoresis analysis

For the expression of different recombinant proteins, single colonies of *E. coli* BL21 (DE3) harboring different recombinant plasmids were used to inoculate Luria–Bertani (LB) medium containing appropriate antibiotics and grown at 37 °C overnight. The saturated culture was diluted 1:100 into fresh LB medium and incubated under the same conditions. When the optical density at 600 nm (OD_600_) of the culture reached about 0.6, all the recombinant proteins expression were induced by 100 μM isopropyl-β-d-thiogalactopyranoside (IPTG) for T_7_ promoter and growth was continued for 4 h at 30 °C. Cells were collected from 5 mL of bacteria cultures by centrifugation and washed with sterile distilled water. The washed pellets were suspended in 500 μL phosphate buffer saline buffer (pH 7.5) and subject to ultrasonication. The cell lysates were centrifuged and the supernatant obtained was mixed with 5× sodium dodecyl sulfate (SDS) sample buffer, heated at 100 °C for 10 min and then analyzed by SDS-polyacrylamide gel electrophoresis (PAGE) according to standard protocols.

### Shake-flask cultivation

To evaluate the ability of glycolate production in different engineered strains, shake-flask experiments were carried out in triplicate series of 250 mL Erlenmeyer flasks containing 50 mL medium with appropriate antibiotics. The medium comprises 14 g/L K_2_HPO4·3H_2_O, 5.2 g/L KH_2_PO_4_, 1 g/L NaCl, 1 g/L NH_4_Cl, 0.25 g/L MgSO_4_·7H_2_O, 0.2 g/L yeast extract. For the production of glycolate with xylose as the sole carbon source, *E. coli* strains were grown overnight at 37 °C with shaking in LB broth, and then 1:50 diluted into 50 mL medium with 15 g/L xylose. When the OD_600_ of the culture reached about 0.6, IPTG was added to a final concentration of 0.1 mM and further incubated 24 h at 30 °C for glycolate production. Samples were taken to determine the OD_600_, the concentrations of glycolate, by-product acetate and the residual xylose.

### Fed-batch fermentation

In order to obtain higher glycolate concentrations, fed-batch cultures were performed in a Biostat B plus MO3L fermentor (Sartorius, Germany) containing 1 L of growth medium (9.8 g/L K_2_HPO_4_·3H_2_O, 2.1 g/L citric acid·H_2_O, 0.3 g/L ferric ammonium citrate, 3.0 g/L (NH_4_)_2_SO_4_) that was sterilized at 121 °C for 25 min. Xylose (20 g/L), MgSO_4_·7H_2_O (0.2 g/L) and 1000× trace elements (3.7 g/L(NH_4_)_6_Mo_7_O_24_·4H_2_O, 2.9 g/L ZnSO_4_·7H_2_O, 24.7 g/L H_3_BO_3_, 2.5 g/LCuSO_4_·5H_2_O, 15.8 g/L MnCl_2_·4H_2_O) were filter-sterilized separately and added prior to initiate the fermentation. 50 mL of seed culture was prepared by incubating the culture in shake flasks containing liquid LB medium overnight at 37 °C. During the fermentation process, continuous sterile air was supplied at a flow rate of 2 L/min. The pH was controlled at 7.0 during the whole fermentation via automatic addition of 25% (w/w) ammonia water, and the temperature was controlled at 37 °C. The dissolved oxygen (DO) level was maintained at 20 ± 1% by automatically adjusting stirring rate from 300 to 700 rpm. Antifoam was added to prevent frothing if necessary.

When the 20 g/L initial xylose was deleted, 60% (w/w) xylose was begun to feed into the fermentor to maintain the residual xylose below 5 g/L. The supply rate of 60% (w/w) xylose was adjusted according to the concentrations of residual xylose determined every 2 h. When the culture was reached to an OD_600_ of about 10, the temperature was switched to 30 °C, IPTG was added to the culture at a concentration of 0.1 mM and further incubated 48 h for glycolate production. Samples of the fermentation broth were removed at appropriate intervals to determine the OD_600_, the concentrations of glycolate and by-product acetate.

### Analytic methods

Culture samples were taken at several times throughout the experiments. The cell concentration was assayed by measuring optical density of the culture at 600 nm (1 OD unit = 0.36 g_CDW_/L). (Cary 50 UV–Vis, Varian). Metabolites in the culture supernatant were analyzed by an Agilent 1200 Infinity series HPLC system equipped with an Aminex HPX-87H (Bio-Rad, Hercules, CA) column (300 × 7.8 mm). All samples were filtered through 0.22 μm syringe filter. Ultrapure water with 5 mM H_2_SO_4_ was used as the eluent at a flow rate of 0.5 mL/min. The oven temperature was maintained at 55 °C. Concentrations of xylose, glycolate and the by-product acetate were calculated based on standard curves.

## Abbreviations

*C. crescentus*: *Caulobacter crescentus*
*E. coli*: *Escherichia coli*
IPTG: isopropyl β-d-thiogalactoside
HPLC: high performance liquid chromatography
PPP: pentose phosphate pathway
TCA cycle: tricarboxylic acid cycle
DO: dissolved oxygen
SDS-PAGE: sodium dodecyl sulfate-polyacrylamide gel electrophoresis
